# Remediating doctors’ performance to restore patient safety: a realist review protocol

**DOI:** 10.1136/bmjopen-2018-025943

**Published:** 2018-10-28

**Authors:** Tristan Price, Nicola Brennan, Jennifer Cleland, Linda Prescott-Clements, Amanda Wanner, Lyndsey Withers, Geoff Wong, Julian Archer

**Affiliations:** 1 Medicine and Dentistry, University of Plymouth, Plymouth, UK; 2 Institute of Education for Medical and Dental Sciences, School of Medicine, Dentistry and Nutrition, University of Aberdeen, Aberdeen, UK; 3 Education, Royal College of Veterinary Surgeons, London, UK; 4 NIHR Collaboration for Leadership in Applied Health Research and Care South West Peninsula (PenCLAHRC), University of Plymouth, Plymouth, UK; 5 Patient Partner, University of Plymouth, Plymouth, UK; 6 Nuffield Department of Primary Care, Health Sciences, University of Oxford, Oxford, UK

**Keywords:** remediation, medical performance, patient safety

## Abstract

**Introduction:**

Underperformance by doctors poses a risk to patient safety. Remediation is an intervention designed to remedy underperformance and return a doctor to safe practice. Remediation is widely used across healthcare systems globally, and has clear implications for both patient safety and doctor retention. Yet, there is a poor evidence base to inform remediation programmes. In particular, there is a lack of understanding as to why and how a remedial intervention may work to change a doctor’s practice. The aim of this research is to identify why, how, in what contexts, for whom and to what extent remediation programmes for practising doctors work to support patient safety.

**Methods and analysis:**

Realist review is an approach to evidence synthesis that seeks to develop programme theories about how an intervention works to produce its effects. The initial search strategy will involve: database and grey literature searching, citation searching and contacting authors. The evidence search will be extended as the review progresses and becomes more focused on the development of specific aspects of the programme theory. The development of the programme theory will involve input from a stakeholder group consisting of professional experts in the remediation process and patient representatives. Evidence synthesis will use a realist logic of analysis to interrogate data in order to develop and refine the initial programme theory into a more definitive realist programme theory of how remediation works. The study will follow and be reported according to Realist And Meta-narrative Evidence Syntheses—Evolving Standards (RAMESES).

**Ethics and dissemination:**

Ethical approval is not required. Our dissemination strategy will include input from our stakeholder group. Customised outputs will be developed using the knowledge-to-action cycle framework, and will be targeted to: policy-makers; education providers and regulators, the National Health Service, doctors and academics.

**PROSPERO registration number:**

CRD42018088779.

**Strengths and limitations of this study**

- The first realist review of doctor remediation.
- A realist methodology that will address the question of why, how, in what contexts, for whom and to what extent remediation programmes for practising doctors work to restore patient safety.
- Meaningful stakeholder input, including patient and public involvement, throughout the review will support the development and dissemination of contextually sensitive strategies for remediating underperformance in medicine.

- Based on secondary data generated from existing literature, therefore is limited by existing literature and its quality.

## Introduction and background

The real human cost of medical underperformance is difficult to measure, but it is estimated that nearly 12 000 patients die in England each year as a result of preventable medical errors.^1^ Yet, the true societal costs when things go wrong are unknown. Incompetent doctors (of which there are relatively few) need to be stopped from practising, but there is a wider and harder problem to solve that will improve medical practice: doctors who underperform.

Doctors can experience performance issues at any stage in their careers and for many different reasons. Performance concerns are often complex, involving multifactorial issues encompassing knowledge, skills and professional behaviours.^2–4^ To ensure patient safety, it is vital that if there are questions about the performance of a doctor they are identified quickly and, where appropriate, support is provided for the practitioner through remediation.^5–7^

Remediation is an intervention, or a range of interventions, that seek to return a doctor to safe practice in response to identified underperformance.^8 9^ Remedying underperformance is a matter of patient safety and is both a practical and a financial imperative. In the UK, it is estimated that it costs around £250 000 to train a doctor to the point of graduation from medical school, rising to £500 000 at the point when a doctor completes specialty training.^10^ Added to this is the cost of litigation; the National Health Service (NHS) paid out more than £1.4 billion in medical negligence claims in 2015/2016 alone, up from £1.2 billion the year before.^11^ Moreover, in the UK, the number of doctors entering the profession is not keeping pace with projected levels of demand, particularly in certain specialties (general practitioners, psychiatrists and specialists in long-term conditions) and in particular geographical locations.^12^ This trend towards an imbalance in the medical workforce is global in scope and exists across developing and developed countries.^13 14^ Given that recruiting and training a sufficient supply of qualified doctors, within the necessary specialties, is a huge investment for any healthcare system, remedying underperformance where possible will be an important component of a sustainable healthcare policy.

Despite the importance of remediation and its prevalence across healthcare systems globally, relatively little is known about how it works and the extent to which it works. A 2009 systematic review by Hauer *et al* on the remediation of practising doctors reported that there is ‘surprisingly little evidence to guide remediation in medical education at all levels’ (Hauer *et al*, p1827).^15^ A more recent systematic review by Cleland *et al* in 2013, on the remediation of medical students and doctors in training, found that ‘rigorous approaches to developing and evaluating remediation interventions are required’ (Cleland *et al*, p242).^16^ The Cleland *et al* review also found that few of the studies that were included reported having informed their approaches with relevant theory.

A further weakness with the existing evidence base for remediation is that it does not sufficiently inform the development of remediation programmes. As noted by Cleland *et al*, ‘we do not know what types of support work, or how much extra teaching is critical… we cannot delineate precisely what works, and why, in remedial interventions’ (Cleland *et al*, p248).^16^ In other words, in order to design high-quality remediation interventions, it is fundamental to understand how the remediation of doctors is supposed to work, for whom and the contexts that lead to different outcomes.

Remediation covers a broad array of interventions, occurs across a range regulatory jurisdictions, in different settings within those jurisdictions and at different stages of a doctor’s career. As such, there is a clear need for research that builds theoretically rich explanations of how remediation works, and does so in such a way that is appreciative of the varying circumstances in which remediation occurs.^17^ Theory-led research is important because it is able to deliver findings at a level of abstraction whereby they are transferable to a range of interventions, while being close enough to actual practice to be relevant to those who plan and deliver remedial interventions.^18^

## Methods and analysis

### Research questions

The overarching aim of this research is to identity how and why remediation interventions work to improve the performance of doctors. Central to realist methodology (described next) is an acknowledgement that the contexts surrounding a remedial intervention, and the way in which a remedial intervention changes the context, will determine the success or otherwise of a remedial programme. Accordingly, the main research question guiding this review is:

Why, how, in what contexts, for whom and to what extent do remediation programmes for practising doctors work to support patient safety?

This research question is operationalised into two main objectives:

1. To conduct a realist review of the literature to ascertain why, how, in what contexts, for whom and to what extent do remediation programmes for practising doctors work to support patient safety.
2. To provide recommendations on tailoring, implementation and design strategies to improve remediation interventions for doctors.

### Realist review

The research question will be addressed by using a realist approach to evidence synthesis, also known as a realist review. A realist review is rooted in the philosophy of realism and seeks to develop theories about how an intervention works to produce its effects. Central to the realist review approach is the generative model of causality which holds that to infer a causal outcome between any two events requires an understanding of the ‘causal mechanisms (M) that connect them and the context in which relationships occur’.^19^ Essentially, this means developing and then interrogating a theory, or theories, about how remediation interventions work.

The context in which an intervention occurs is central to a realist explanation of how that intervention works to produce its effects. Context may relate to the specific structures or the environment surrounding an intervention, or to characteristics of those individuals delivering or receiving the intervention.^19^ However, the realist approach seeks not to simply list all the contextual factors surrounding an intervention, but to establish which of the contextual factors are necessary to explain how the intervention produces the outcome. That is where the concept of the mechanism comes into play. Mechanisms are the way in which a programme’s resources or opportunities interact with the reasoning of individuals and lead to changes in behaviour. Mechanisms are usually hidden in that they are often not labelled as an official component of the programme, but can be deduced through research into how those types of programme work for particular people in particular circumstances to produce the desired outcome.^20^

A key part of the realist review approach is developing a programme theory, or theories. A programme theory is a description and/or a diagram that depicts how the intervention is supposed to work to produce its effects. Any programme theories developed should be specified at the middle-range level of abstraction—in other words, specified in such a way that permits them to be ‘tested’ against the empirical data from documents included in the realist review. Importantly, a realist review starts and ends with a programme theory; the programme theory is developed, interrogated and refined through an iterative process of collecting and analysing data from a variety of sources.

Realist reviews are, therefore, particularly suited to understanding complex and multifaceted interventions like remediation, where a variety of approaches are employed within different contexts.

### Study design

Pawson *et al* have developed five practical steps to conduct realist reviews that will guide the research process in this study.^21^ It is important to note that although these stages are numbered sequentially, realist reviews are iterative by nature and therefore there will be some movement between stages as the research progresses. The study findings will be written up according to the Realist And Meta-narrative Evidence Syntheses—Evolving Standards (RAMESES) quality and publication standards.^22^ This protocol is reported according to Preferred Reporting Items for Systematic Reviews and Meta-Analyses Protocols guidelines.^23^

#### Step 1: locate existing theories

This stage involves identifying the existing theories that explain how remediation is supposed to work. To identify these theories, we shall search relevant personal libraries of members of the review team. The research fellow (TP) will also undertake informal searches of the existing literature, informed by previous research into remediation undertaken by members of the review team (TP, JA, NB and JC) to develop the funding application for this review. We shall also iteratively consult with recognised experts in the remediation field, some of whom are coapplicants on this review (JC and LP-C), and others with whom we collaborate. In addition, we shall undertake relatively open searches of databases such as Google Scholar using keywords such as ‘remediation’. The initial programme theory will be developed by TP through identifying some of the key activities that occur in remediation programmes and any existing explanations of how such activities work to bring about changes in doctor performance related to areas such as knowledge, skills, attitudes, professional behaviours or the workplace environment.

We have established a stakeholder group to help develop the initial programme theory and refine the theory as the review progresses. The stakeholder group comprises a variety of professionals working within medicine (including doctors who have undergone remediation) and non-clinicians within clinical settings, representatives from doctor and patient groups, the medical regulator (the General Medical Council (GMC)) and the National Clinical Assessment Service (NCAS). NCAS is an important collaborator in this review as they are the NHS body that provides advice, support and assessment services to help resolve concerns about the professional practice of doctors in the UK (as well as dentists and pharmacists).

The stakeholder group will assist with the development of the initial programme theory and its subsequent refinement. Stakeholder meetings will be convened every three months and will be used to develop the initial programme theory as well as refining the programme theory through the duration of the review. At the first meeting, we shall seek to ascertain the stakeholders’ broad perspectives on the review questions and their own experience of remediation. At subsequent meetings, we shall present to them our emerging research findings, using their feedback to further refine the programme theory. In addition to supporting the research to develop and refine the programme theory, the stakeholder group will also have a role in aiding the dissemination of the review findings to achieve maximum impact.

#### Step 2: search strategy

##### Formal searches

Conducting a realist review is an iterative process. An initial search strategy has been developed that will seek to catch all of the existing literature on the remediation of doctors to help inform the programme theory on how remediation is meant to work to produce improved performance in doctors. The search strategy has been developed and piloted with an information specialist (AW) who is part of the core research team. Initial search terms were developed and tested against a ‘gold standard’ set of representative articles identified by subject experts. The initial search has been designed to capture a broad range of literature: all articles or studies that report on the remediation (ie, the remedy of identified underperformance) of practising doctors (ie, medical professionals who have graduated from medical school and hold a licence to practice medicine). The search strategy will include:

- Searching electronic databases including using key word searches related to the remediation of practising doctors, including: Embase, MEDLINE, Cumulative Index to Nursing and Allied Health Literature (CINAHL), PsycINFO, Educational Resources Information Centre (ERIC), Database for Abstracts of Reviews and Effects (DARE), Applied Social Sciences Index and Abstracts (ASSIA) and Health Management Information Consortium (HMIC).
- Forward and backward citation searches of all articles that are included.
- Making contact with authors if necessary.
- Searching the grey literature, particularly of those bodies that deliver or plan remediation interventions. Google, OpenGrey and HMIC will also be searched.

As the review progresses, the searching will become more focused on key areas of the programme theory.

##### Additional searches

A vital part of conducting a realist review involves searching for additional data to explain particular parts of the programme theory. Therefore, more searches will be conducted in any such identified areas as the review progresses. Based on our understanding of remediation to date, these could include areas like feedback on performance,^24–26^ reflection^27 28^ and development of insight.^29–32^ These additional topics will increase the quantity of relevant data available for us to test the programme theory. The searches will be developed, piloted and refined by the core research team with the help of the information specialist. These searches will differ from the ‘formal searches’ outlined above through being more exploratory and purposive, and will emanate from a range of different disciplines. Each additional search instigated, along with the inclusion and exclusion criteria, will be discussed by the core research team.

#### Step 3: study selection criteria and procedures

Our document selection process will be as follows. Screening of documents from our search(es) will be piloted with small samples being screened by two members of the research team (TP and NB), until high levels of agreement are reached. Full screening will be conducted by one member of the research team (TP). A random sample of 10% of the citations identified through the formal searches will be reviewed independently by NB for quality assurance purposes. Disagreement will be resolved through discussion with the whole research team.

Article selection is based on relevance, in other words the extent to which an article can contribute to the development of the programme theory.^18 19^ Accordingly, at the initial stage of the review, we may include any documents that contain relevant data—for example, original studies of different types, commentaries, systematic reviews and grey literature reports and guidance documents.

#### Step 4: extracting and organising data

The iterative process of realist reviewing dictates a different method for extracting data than is used in a more conventional systematic review, using note taking and annotation as opposed to a standard data extraction form. Documents will be examined for data on how a remediation intervention is supposed to work. The synthesis of evidence will begin with conceptual coding using NVivo qualitative data management software.^33^ As the review progresses, these conceptual codes will be analysed to develop context-mechanism-outcome configurations (CMOC) (see Step 5: data synthesis). Data on the characteristics of the documents will be extracted separately into an Excel spreadsheet. Data extraction will be carried out by TP.

#### Step 5: data synthesis

Data analysis will involve the use of a realist logic analysis with the goal of using the data from the literature (ie, sources) to further develop the initial programme theory. Analysis requires interpretation and judgement of data. Data coding will be deductive (informed by our initial programme theory), inductive (from the data within documents) and retroductive (where inferences are made based on interpretations of the data within documents about underlying causal processes—ie, mechanisms). We shall use a series of questions about the relevance and rigour of content within sources as part of our process of analysis, as set out in box 1.

Data to inform our interpretation of the relationships between contexts, mechanisms and outcomes will be sought not just within the same source, but across sources (eg, mechanisms inferred from one source could help explain the way contexts influenced outcomes in a different source). Synthesising data from different sources is often necessary to compile CMOCs, since not all parts of the configurations will always be articulated in the same source.

Box 1 **Data analysis in realist reviews**

**Questions to guide data analysis in a realist review:**

**Relevance**

- Are there sections of text within this source that are relevant to programme theory development?

**Rigour—judgements about trustworthiness**

- Are these data sufficiently trustworthy to warrant making changes (if needed) to any aspect of the programme theory?

**Interpretation of meaning**

- If the section of text is relevant and trustworthy enough, do its contents provide data that may be interpreted as functioning as context, mechanism or outcome?

**Interpretations and judgements about context-mechanism-outcome configurations**

- For the data that have been interpreted as functioning as context, mechanism or outcome, which context-mechanism-outcome configuration (CMOC) (partial or complete) does it belong to?
- Are there further data to inform this particular CMOCs contained within this source or other sources? If so, in which other documents?
- How does this particular CMOC relate to other CMOCs that have already been developed?

**Interpretations and judgements about programme theory**

- How does this particular (full or partial) CMOC relate to the programme theory?
- Within this same source are there data which informs how the CMOC relates to the programme theory? If not, are there data in other sources? Which ones?
- In light of this particular CMOC and any supporting data, does the programme theory need to be changed?

Within the analytic process set out in box 1 (adapted from Papoutsi *et al* 2017),^34^ we shall use interpretive cross-case comparison to understand and explain how and why observed outcomes have occurred, for example, by comparing interventions where remediation has been ‘successful’ against those which have not, to understand how context has influenced reported findings. When working through the questions set out, where appropriate we shall use the following forms of reasoning to make sense of the data:

- Juxtaposition of data: for example, where data about behaviour change in one source enable insights into data about outcomes in another source.
- Reconciling of data: where data differ in apparently similar circumstances, further investigation is appropriate in order to find explanations for why these differences have occurred.
- Adjudication of data: on the basis of methodological strengths or weaknesses.
- Consolidation of data: where outcomes differ in particular contexts, an explanation can be constructed of how and why these outcomes occur differently.

During the review, we shall move iteratively between the analysis of particular examples, refinement of the programme theory and further iterative searching for data to test particular theories. The final realist programme theory will be presented in a diagram and through a narrative description of CMOCs.

### Patient and public Involvement

Patient and public involvement (PPI) has been central to the design of this study and will continue to be a meaningful component of this review. Discussions with an existing PPI forum, attached to ongoing research collaborations, had drawn attention to the lack of research on remediation and its implications for patient safety. These concerns helped define the research focus and forum members provided critical feedback on various iterations of the funding proposal. A University of Plymouth patient partner (LW) is a coapplicant on the study and there is lay representation at all stages of the research, including dissemination, through the stakeholder group.

## Discussion

### Importance of the research

The proposed research will make an empirical contribution to the existing body of knowledge by developing a transferable realist programme theory of how remediation of doctors works, for whom and in what contexts. Achieving this type of understanding will also enable us to develop recommendations to support the optimal tailoring, design and implementation of remediation interventions for underperforming doctors in order to support patient safety.

This research will generate new knowledge about a poorly understood area of healthcare delivery that directly affects the standards of care received by patients. It is thus consistent with a focus on improving the quality and the organisation of health services, in this instance within the specific area of improving the design and delivery of remediation programmes.

The research will be carried out with NCAS as a collaborative partner, and will therefore have a direct impact in terms of shaping NCAS remediation programmes in the UK. This collaboration, combined with expert input from our stakeholder group, will ensure that the study will deliver findings that will directly feed into policy and practice development and have international significance. The self-evident importance of doctor performance for patient safety, and the practical, moral, political and financial imperatives of offering underperforming doctors the opportunity to remediate mean that this will be an area of sustained international interest in the area of health services research.

### Dissemination

Our dissemination strategy will build on the participatory approach (involving stakeholders) that we shall develop throughout the preceding stages of the review. We shall work with the representatives from NCAS, who are part of the stakeholder group, to refine our dissemination strategy throughout the study. We shall also seek to engage with other audiences who have a stake in our research.

This dissemination strategy will aim to have impact along three primary trajectories:

#### Instrumental impact

The study will inform and develop the policy and practice of remediation. This refers to the findings of the review itself and our dissemination of review findings to key stakeholders such as NCAS and the GMC in order to provide tangible improvement to the practice of remediation in NHS organisations.

#### Conceptual impact

The study will be the first of its kind to conduct a realist review of remediation and to develop a programme theory of remediation. The systematic reviews that exist on this topic are now dated (2009 and 2013); no one has, as yet, conducted a review of remediation to work out what works, for whom, how, why and in what contexts (ie, a realist review) as proposed by Cleland *et al* in the later review.

#### Capacity building

The networks that are developed through conducting and disseminating the research will enhance the collective technical expertise in the area for further research and development of remediation practices.

We want to ensure that the outputs of this project will be useful to the NHS. To do this, we shall use the knowledge-to-action cycle framework provided by Clearinghouse.^35^ This is a framework that provides knowledge translation resources funded by the Canadian Institute of Health Research. The knowledge-to-action cycle graphically sets out the steps necessary in bridging the knowledge-to-action gap. Specifically, with input from our stakeholder group, this realist review will generate knowledge that will inform the following phases of the knowledge-to-action cycle framework by: producing stakeholder relevant knowledge; adapting knowledge to local context and assessing barriers to knowledge use.

We shall seek to operationalise this framework by:

##### The findings from the review will be submitted for publication to a high-impact peer-reviewed journal

We anticipate that this publication is most likely to impact at an academic level—informing the understanding and theoretical basis of remediation behaviour change interventions.

##### A ‘user guide’ that outlines practical advice to optimise, tailor and implement existing interventions designed to change behaviour through remediation

With this output, we shall aim to impact on the landscape of current remediation provision. This document will be targeted at educational providers and regulators. These include medical schools, Local Education Training Boards and Deaneries, as well as Health Education England, NHS Education Scotland, the NHS, the GMC and NCAS. These bodies are at the delivery end of existing remediation practices that we wish to inform and help improve.

We shall draw on the expertise of the academics and educators within our project team and combine this with the policy expertise of the wider stakeholder group to produce an accessible, relevant and practical guide. This will ensure that it can be used to bring about direct change in policy and remediation practice.

##### User-friendly summaries of the review findings that are tailored to the needs of interested audiences

Stakeholders will be invited to attend presentations on the developing programme theory so that research dissemination can also benefit from their feedback and reflection. In addition to national and regional dissemination, research findings will be presented locally and internationally. Locally, we shall continue to work with researchers across Plymouth through CAMERA’s monthly meetings to share and promote research. At an international level, our established networks in North America and Australasia will continue, allowing international comparisons between practice in the UK and systems for remediating poor performance around the world.

To support PPI beyond the stakeholder group, the research will be summarised in a newly developed website.^36^

## Supplementary Material

Reviewer comments

Author's manuscript
