# Supplementary material for: Remediating doctors’ performance to restore patient safety: a realist review protocol
Source: BMJ Open. 2018 Oct 28;8(10):e025943. doi: 10.1136/bmjopen-2018-025943 (PMC6224734; doi:10.1136/bmjopen-2018-025943)
Supplement: Reviewer comments [file bmjopen-2018-025943.reviewer_comments.pdf]

PEER REVIEW HISTORY

BMJ Open publishes all reviews undertaken for accepted manuscripts. Reviewers are asked to complete a checklist review form (**http://bmjopen.bmj.com/site/about/resources/checklist.pdf**) and are provided with free text boxes to elaborate on their assessment. These free text comments are reproduced below.

ARTICLE DETAILS

**TITLE (PROVISIONAL)**	Remediating doctors' performance to restore patient safety: A realist review protocol
**AUTHORS**	Price, Tristan; Brennan, Nicola; Cleland, Jennifer; Prescott-Clements, Linda; Wanner, Amanda; Withers, Lyndsey; Wong, Geoff; Archer, Julian

This article was not externally reviewed at BMJ Open. Protocols that have been independently assessed prior to submission to BMJ Open are usually fast-tracked to publication on the grounds that further substantial changes will not be possible. This independent assessment will usually be external, independent review for both a substantial grant award from a non-commercial or government funder and ethics approval.
